# Development of a new TaqMan-based real-time RT-PCR assay for the specific detection of bovine kobuvirus

**DOI:** 10.3389/fvets.2022.953599

**Published:** 2022-08-04

**Authors:** Yuelin Liu, Libing Liu, Jinfeng Wang, Ting Wang, Yaxin Gao, Xiaoxia Sun, Wanzhe Yuan, Ruiwen Li, Jianchang Wang

**Affiliations:** ^1^Department of Clinical Veterinary Medicine, College of Veterinary Medicine, Hebei Agricultural University, Baoding, China; ^2^Department of Animal and Plant Quarantine, Technology Center of Shijiazhuang Customs District, Shijiazhuang, China; ^3^Department of Animal Genetics and Breeding, Hebei Mountain Agricultural Technology Innovation Center, Hebei Agricultural University, Baoding, China

**Keywords:** bovine kobuvirus, 3D gene, TaqMan probe, real-time RT-PCR, phylogenetic analysis

## Abstract

Bovine kobuvirus (BKV) is a novel kobuvirus considered to be closely related to calf diarrhea and has become a worldwide epidemic. Currently, the BKV lacks an efficient and convenient detection method to assist the research on BKV prevalence. In this study, a new and specific TaqMan-based real-time RT-PCR for the detection of BKV was developed using the conserved region of the 3D gene. The assay was highly specific for BKV, without cross-amplification with other non-targeted pathogens. The limit of detection of this assay was 10^2^ copies. Standard curves showed a strong linear correlation from 10^2^ to 10^6^ copies of BKV standard RNA per reaction, and the parameters revealed as a slope of −3.54, efficiency of 91.64%, and regression coefficients (R^2^) of 0.998. The assay was also reproducible, with the intra-assay and inter-assay coefficient of variation <1.0%. The newly developed real-time RT-PCR was validated using 243 fecal samples collected from diarrheic or non-diarrheic cattle from nine regions in Hebei province and revealed the positive detection of BKV at a ratio of 19.34% (47/243). Sequencing of partial 3D genes from 13 positive samples and the following phylogenetic analysis demonstrated the reliability of the assay. In conclusion, the newly developed TaqMan-based real-time RT-PCR could be used for the screening and epidemic monitoring of BKV.

## Introduction

Neonatal calf diarrhea (NCD) poses a huge threat to the health of newborn cattle which even leads to death, causing huge economic losses to the cattle industry. Bovine kobuvirus (BKV) belongs to the genus *Kobuvirus* of the Picornaviridae family (1) and is considered to be closely related to calf diarrhea (2). Since the kobuvirus was first discovered in 1989 (3), it has been divided into six species (Aichivirus A-F) (4), which can infect humans (5) and several domestic animals including cattle, sheep, swine, goats, rabbits, cats, and dogs as well as wild canids, boars, and ruminants (1, 6–11). BKV was classified as Aichivirus B (4), and the virus had been detected in diarrheic or non-diarrheic cattle in Europe (10, 12–14), Asia (2, 15, 16), Africa (17, 18), and the Americas (19, 20) since it was first isolated in Japan in 2003 (1). For the bovine clinical diarrhea samples, BKV was always observed to be co-infected with other common pathogens causing diarrhea (15, 21, 22), such as bovine rotavirus A (BRVA), bovine coronavirus (BCoV), and Cryptosporidium. However, it is noteworthy that many studies had demonstrated that BKV may lead to diarrhea in neonatal cattle (2, 15, 16, 20, 23).

Studies focusing on the prevalence of BKV have received widespread attention worldwide, but there is still a lack of an efficient and convenient detection method to assist. At present, the main detection methods for BKV include virus isolation and identification (1), and conventional RT-PCR (8, 16), but none of these methods could cope with the need to quickly detect BKV in a large number of samples. Virus isolation is a definitive diagnosis for BKV infection, however, this assay is usually unfeasible owing to less sensitivity, being a laborious process, and requiring special facilities and skilled personnel, as well as fresh specimens with viable viruses. RT-PCR assays have proved to be very sensitive and specific, but still require time-consuming post-PCR analysis and are prone to cross-contamination. Real-time PCR assay has gained wide acceptance due to its improved rapidity, simplicity, reproducibility, and reduced risk of carry-over contamination (24). Real-time PCR had been widely used for the detection of various pathogens (25–27), which demonstrated great importance in the early diagnosis of diseases, the detection of latent infections, and the epidemiological analysis of the pathogens. Therefore, it is necessary to develop a rapid, simple, sensitive, and specific diagnostic method for BKV infection and surveillance.

In this study, we designed specific primers and TaqMan probe based on the 3D gene of BKV and developed a real-time RT-PCR assay for BKV. Subsequently, the assay was tested on bovine fecal samples collected from 9 different regions in Hebei province for the presence of BKV.

## Materials and methods

### Virus strains and clinical samples

Viral RNA of bovine kobuvirus (BKV, HB-ZJK/2022), bovine rotavirus A (BRVA, HB-LF/2021), bovine viral diarrhea virus (BVDV, C24V), bovine coronavirus (BCoV, HB-SJZ/2021), bovine enterovirus (BEV, HB-BD/2021), bovine torovirus (BToV, HB-HS/2021), and viral DNA of bovine adenovirus 7 (BAdV 7, HB-YS531/2021) were preserved in our laboratory.

In total, 243 bovine fecal samples were collected from nine regions of Hebei between May 2021 and May 2022. Among the 243 fecal samples, 162 (66.67%) were collected from cattle presenting diarrhea while 81 (33.33%) were collected from non-diarrheic cattle. Regarding the age, 90 diarrheic fecal samples were collected from calves younger than 3 months while 72 diarrheic fecal samples were collected from adult cattle; all 81 non-diarrheic fecal samples were from adult cattle. The details of all samples are listed in Table 1.

**Table 1:** Detailed information on the collected bovine fecal samples.

**Locations**	**Number of** ** samples**	**Months of** ** cattle**	**Clinical symptoms**
Shijiazhuang	9	<3	Diarrhea
	27	<18	Diarrhea
	7	<18	Non-diarrhea
Baoding	18	<3	Water-like diarrhea
	23	<18	Diarrhea
	6	<18	Non-diarrhea
Zhangjiakou	21	<3	Diarrhea
	13	<18	Diarrhea
Hengshui	7	<3	Water-like diarrhea
	7	<18	Diarrhea
Handan	10	<3	Water-like diarrhea or bloody diarrhea
Langfang	23	<3	Water-like diarrhea
Xingtai	2	<3	Water-like diarrhea or bloody diarrhea
	2	<18	Diarrhea
Chengde	48	<18	Non-diarrhea
Cangzhou	20	<18	Non-diarrhea
Total	243	

### RNA extraction

To extract RNA, 200 mg of normal fecal or 200 μl of watery fecal were added to phosphate-buffered saline (PBS, pH7.4) and homogenized as a 10% (w/v) suspension. The homogenization was centrifuged for 5 min at 10,000 g at 4°C. According to the manufacturer's instructions, the viral RNA was extracted from 200 μl of the supernatant using the TIANLONG Magnetic Viral DNA/RNA kit (Tianlong, XiAn, China) with the Automatic Nucleic Acid Extraction Instrument (np968-c, Tianlong, XiAn, China). All the extracted viral nucleic acids were tested for concentration and purity using ND-2000c spectrophotometer (NanoDrop, Wilmington, United States) and then used immediately or stored at −80°C until use.

### Generation of RNA standard

First-strand cDNA of BKV was generated using the PrimeScript first-strand cDNA Synthesis Kit (TaKaRa, Dalian, China) with viral RNA of BKV, and the cDNA was used as a template in the following PCR. The PCR primers (3D-F and 3D-R) were designed based on the conserved sequence of 3D gene of BKV (Table 2) and used to amplify the 1,383 bp fragment. The PCR product was purified using the Zymoclean Gel DNA Recovery Kit (Zymoresearch, United States), cloned into a pGEM-T Easy vector (Promega, Madison, United States), and named pGEM-T-BKV-3D. The constructed plasmid was confirmed by DNA sequencing (Tsingke Biotechnology Co., Ltd., Beijing, China) and performed *in vitro* transcribed using RiboMAX Large Scale RNA Production System-T7 (Promega, Madison, United States) to produce a standard RNA of BKV. The standard RNA was quantified using ND-2000c spectrophotometer (NanoDrop, Wilmington, United States) and calculated the copy number of RNA molecules by a previously published formula (28). To generate standard RNA of BKV, 10-fold serial dilutions ranging from 1.0×10^6^ to 1.0×10^0^ copies/μl were made with RNase-Free ddH_2_O and used.

**Table 2:** Sequences of the primers and probe for BKV RT-PCR and real-time RT-PCR assays.

**Assay**	**Primers and probe**	**Sequence 5^′^-3^′^**	**Amplicon size (bp)**	**References**
PCR	3D-F	CTGATCATACCAGGCCCGGAA	1,383	This study
	3D-R	GCGAAGCTGGAGATATTCATAAGG		
	10f	GATGCTCCTCGGTGGTCTCA	631	Yamashita et al. (1)
	10r	GTCGGGGTCCATCACAGGGT		
real-time RT-PCR	BKV-F	CACTCCCGCCAACAAAGGT	87	This study
	BKV-R	TCATCTGGAACAAACCATCGTT		
	BKV-P	FAM-TCTTCCACTCTCTACGACGTCACCTTCCTC-BHQ1		

### Design of primers and TaqMan probe

The 3D gene was set as the target of the real-time RT-PCR assay because it is highly conserved among the different BKV strains (1, 16). The complete sequences of 3D gene (Accession numbers: MK080265, MN336260, MW605074, MZ603734, KT003671, KY407744, ON075050, ON075051, ON075052, ON075053, ON075054, ON075055, ON075056) from different countries were obtained from the GenBank and aligned using the DNASTAR (DNASTAR, Madison, United States) to identify the conserved region. The primers and TaqMan probe were designed based on the conserved region using the Primer Express software (version 3.0) (Applied Biosystems, Foster City, United States) referring to CHZ/China strain (MK080265.1). The primers and TaqMan probe were synthesized by a commercial company (Generay, Shanghai, China) and are shown in Table 2.

### Optimization of the real-time RT-PCR assay

All real-time RT-PCR assays were performed on ABI Quant Studio 5 (Applied Biosystems, Foster City, CA, United States). Each reaction consisted of 2×PerfectStart Probe One-Step qPCR SuperMix (TransGen Biotech, Beijing, China) 12.5 μl, TransScript Probe One-Step RT/RI Enzyme Mix 0.5 μl, 1 μl of standard RNA, in addition to different volumes of primers (0.6, 0.8, 1.0, 1.2, 1.4, 1.6 μl, 10 μmol/L) and probe (0.2, 0.4, 0.6, 0.8, 1.0, 1.2 μl, 10 μmol/L), which were used to optimize the assay and made up to 25 μl with the RNase-Free ddH_2_O. The reaction condition was set as follows: 45°C for 5 min and 94 °C for 30 s, followed by 40 cycles of 94°C for 5 s and 55–62°C for 30 s, which were used to confirm the optimal amplification conditions. All optimization assays were performed in parallel with the same standard RNA as the template.

### Analytical specificity and sensitivity analysis

The viral RNA or DNA of 7 important viruses that cause diarrhea in cattle including BKV, BRVA, BVDV, BCoV, BToV, BEV, and BAdV 7 were used for the analytical specificity analysis. Furthermore, the nucleic acid extracted from BKV-negative feces was used as negative control and ddH_2_O was used as non-template control. The analytical specificity analysis was repeated three times. The analytical sensitivity of the assay was confirmed by amplifying 1.0×10^6^-1.0×10^0^ copies/μl of the standard RNA of BKV. The analytical sensitivity analysis was repeated three times and in quintuplicate parallel for each concentration of standard RNA. In addition, both analyses were verified for the presence of cross-contamination by adding ddH_2_O as non-template control (NTC).

### Generation of standard curve

The standard curve of the developed real-time RT-PCR assay was generated by testing 10-fold serial dilutions of standard RNA with concentrations ranging from 1.0×10^6^ to 1.0×10^0^ copies/μl using ABI Quant Studio 5. The analysis was repeated three times and the PCR amplification efficiency (E), standard curve slope, and correlation coefficient (R^2^) were calculated as described previously (29).

### Reproducibility of the real-time RT-PCR assay

The reproducibility of the developed real-time assay was evaluated using the coefficient of variation (CV). The CV was calculated with a threshold cycle (Ct), which was carried out on five different days (inter-assay) and in quintuplicate (intra-assay) using the 1.0×10^6^, 1.0×10^4^, and 1.0×10^2^ copies of standard RNA as a template in the real-time RT-PCR.

### Detection of BKV in the clinical samples

Viral RNA extracted from 243 bovine fecal samples were detected for BKV by the developed real-time RT-PCR and a previously described conventional RT-PCR in parallel (1). Three microliters of extracted RNA from all samples were used as templates. In the developed real-time RT-PCR assay, samples with a Ct value <35 were judged positive. For samples with a Ct value between 35 and 40, the nucleic acid was re-extracted and re-tested. If they showed a typical amplification curve and a Ct value <40, it was judged as positive; otherwise, it would be judged as negative.

### Sequence and phylogenetic analysis

At least two BKV RNA-positive samples per region were used for sequence and phylogenetic analysis to verify the accuracy of detection results. For the selected BKV RNA-positive samples, a 631 bp fragment was generated by a previously published RT-PCR protocol with the primers 10f and 10r (1). All PCR products were sequenced directly in both directions by a commercial company (Tsingke Biotechnology Co., Ltd., Beijing, China), and numerous sequences of BKV 3D gene were retrieved from the GenBank. Both the obtained sequences and BKV reference sequences were cropped to 552 bp according to a previous study (16) using SeqMan software (version 7.0; DNASTAR Inc., WI, United States), and then homologies were analyzed of all sample strains using the MegAlign software (version 7.0; DNASTAR Inc., WI, United States). Both the positive sample sequences and reference sequences were performed with a multiple sequence alignment, and a phylogenetic tree with bootstrap support was constructed with classical Aichivirus A (AB040749) and porcine kobuvirus (EU787450) as outgroups by using the MEGA 6.05 software.

## Results

### Optimization of the real-time RT-PCR

The optimal volumes of primers, probes, and the annealing temperature were defined based on the presence of a plateau period and minimum Ct value. The following parameters were determined as the optimal reaction conditions: 1 μl of each primer (10 μmol/L) and 0.4 μl of probe (10 μmol/L) for the amplification systems, and 56 °C for 30 s for the annealing temperature and extension time.

### Analytical specificity and sensitivity of the real-time RT-PCR

**Figure 1:**
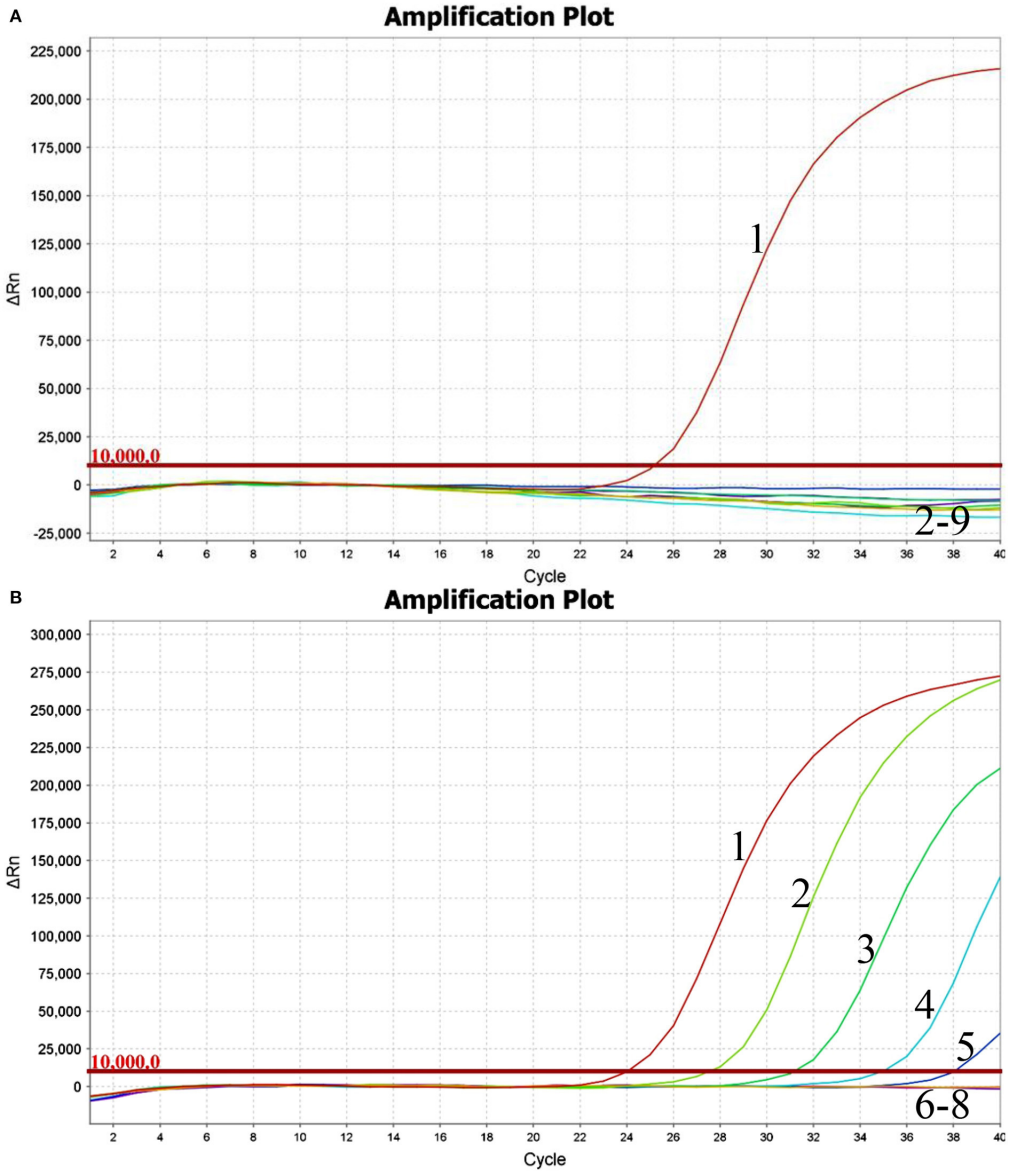
Performance of BKV-specific real-time RT-PCR assay. Only the BKV RNA was amplified, and the limit of detection of the assay was 1.0×10^2^copies. **(A)** Analytical specificity of the real-time RT-PCR assay evaluating the common viruses causing diarrhea in cattle. Line 1, BKV; line 2, BRVA; line 3, BVDV; line 4, BCoV; line 5, BToV; line 6, BEV; line 7, BAdV 7; line 8, negative control; line 9, ddH_2_O. **(B)** Analytical sensitivity of the real-time RT-PCR assay using a dilution range of 1.0×10^6^-1.0×10^0^copies of BKV standard RNA. Line 1, 1.0×10^6^copies; line 2, 1.0×10^5^copies; line 3, 1.0×10^4^copies; line 4, 1.0×10^3^copies; line 5, 1.0×10^2^copies; line 6, 1.0×10^1^copies; line 7, 1.0×10^0^copies; line 8, ddH_2_O.

Using the viral RNA or DNA, negative control, and ddH_2_O as templates, the typical amplification curve was only observed for BKV (Figure 1A). Three independent reactions showed similar results, confirming the good stability of the analytical specificity of the developed assay.

Using the BKV standard RNA ranging from 1.0×10^6^ to 1.0×10^0^ copies/μl as a template, the typical amplification curves were always observed for the 10^6^-10^2^ copies, while the amplification curves were never observed for the 10^1^, 10^0^ copies, and ddH_2_O (Figure 1B). Similar results were obtained in quintuplicate parallel of all three independent assays. The limit of detection of the developed real-time RT-PCR was determined to be 100 copies, suggesting that the assay showed good sensitivity for the detection of BKV RNA.

### Standard curve

**Figure 2:**
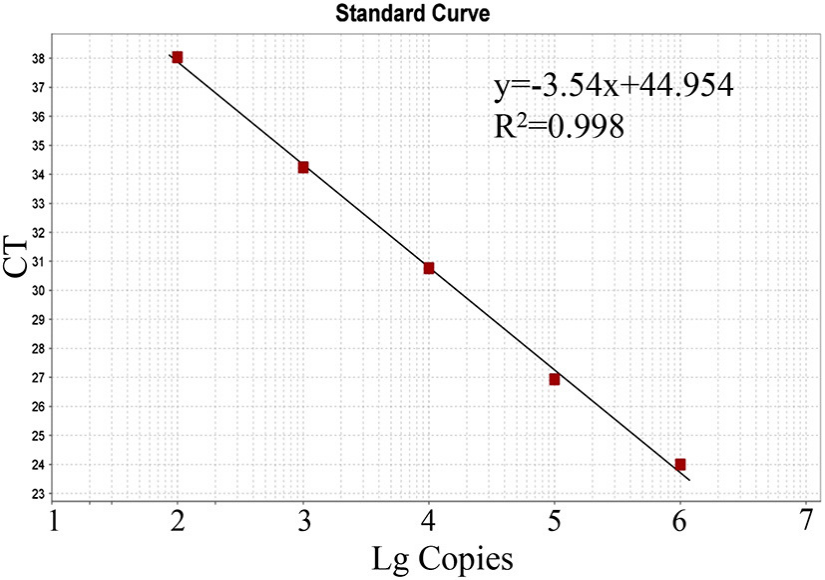
Standard curve of the real-time RT-PCR for BKV based on serial dilutions of the standard RNA. The assays were linear over a 10^2^-10^6^ dilution range with R^2^ values of 0.998 and reaction efficiency of 91.64%.

The generated standard curve showed a strong linear correlation from 10^2^ to 10^6^ copies per reaction and the parameters revealed as a slope of −3.54, efficiency of 91.64%, and R^2^ of 0.998 (Figure 2).

### Reproducibility of the real-time RT-PCR

The assay reproducibility was assessed based on the CV of inter-assay and intra-assay (Table 3). The intra-assay CVs of 1.0×10^6^, 1.0×10^4^, and 1.0×10^2^ copies of BKV standard RNA were 0.33, 0.37, and 0.38%, and the inter-assay CVs were 0.55%, 0.33, and 0.34%, respectively, which showed reproducibility of the developed real-time RT-PCR.

**Table 3:** Reproducibility of the real-time RT-PCR evaluated with standard RNA of BKV.

**Standard RNA**	**Copy number** ** (copies/μl)**	**Intra-assay**		**Inter-assay**	
		**Ct ($\bar{x}$ ±s)**	**CV/%**	**Ct ($\bar{x}$ ±s)**	**CV/%**
BKV 3D RNA	1.0 ×10^6^	25.25 ± 0.08	0.33	24.92 ± 0.14	0.55
	1.0 ×10^4^	32.04 ± 0.12	0.37	32.36 ± 0.11	0.33
	1.0 ×10^2^	38.15 ± 0.15	0.38	38.06 ± 0.13	0.34

### Evaluation of real-time RT-PCR with clinical samples

Testing of 243 bovine fecal samples revealed the positive detection of BKV in 47 samples in the developed real-time RT-PCR (Table 4), while only 42 samples were detected to be BKV positive in conventional RT-PCR. Further analysis demonstrated that all the 42 samples were also BKV positive in real-time RT-PCR, and 5 samples with inconsistent results in the two methods showed higher Ct values (35.43–37.57), which contained low amounts of BKV RNA. From the perspective of clinical symptoms, 36 (26.22%) were obtained from cattle presenting diarrhea, and 11 (13.58%) were obtained from non-diarrheic cattle. According to the Ct values and the generated standard curve, the level of BKV viral RNA in 36 symptomatic BKV-positive cattle was 6.71×10^6^ ± 3.76×10^6^ copies, while the level of viral RNA in 11 non-symptomatic BKV-positive cattle was 3.04×10^3^ ± 1.58×10^3^ copies. From the perspective of cattle age, 27 (30.00%) were obtained from calves younger than 3 months, and 20 (13.07%) were obtained from adult cattle.

**Table 4:** BKV detection results of clinical samples by the developed real-time RT-PCR.

**Age group**	**Clinical symptoms**				**Animals evaluated**	
	**Diarrhea**		**Non-diarrhea**		
	**Total**	**Positive (%)**	**Total**	**Positive (%)**	**Total**	**Positive (%)**
Calves	90	27 (30.00%)	0	0	90	27 (30.00%)
Adult	72	9 (12.50%)	81	11 (13.58%)	153	20 (13.07%)
Total	162	36 (22.22%)	81	11 (13.58%)	243	47 (19.34%)

### Sequence and phylogenetic analysis

**Figure 3:**
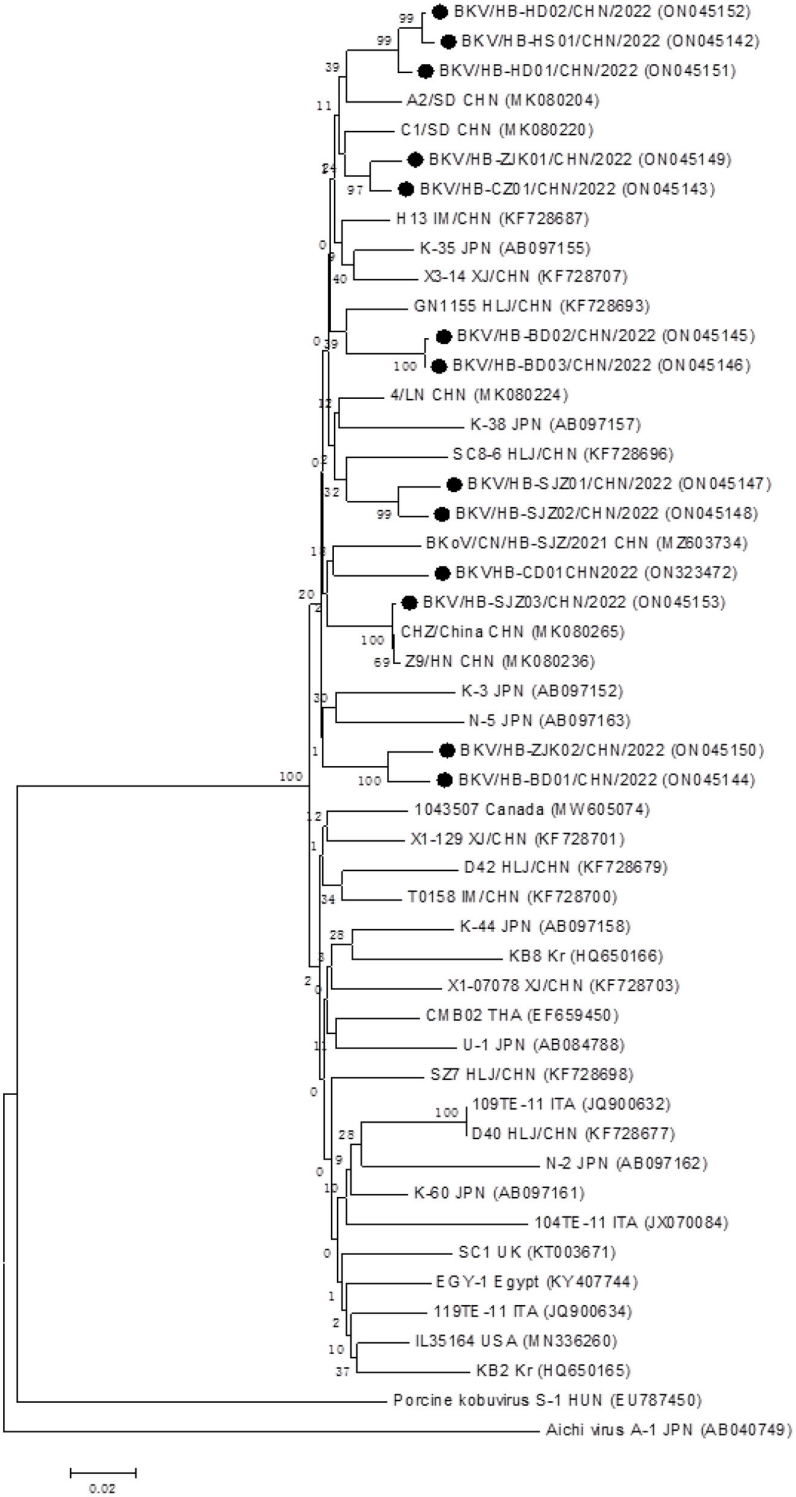
Phylogenetic analysis of the BKV strains based on the nucleotide sequences of their 552 bp partial 3D genes. The phylogenetic tree was constructed by the neighbor-joining clustering method with 1,000 bootstrap replicates, using MEGA version 6.05 software. Black circles referred to Hebei strains identified in this study. All accession numbers used in this study had been labeled in the figure.

In total, 13 BKV RNA-positive samples were sequenced and used for further phylogenetic analysis, sourced from Shijiazhuang (*n* = 3), Baoding (*n* = 3), Zhangjiakou (*n* = 2), Handan (*n* = 2); Hengshui (*n* = 1), Cangzhou (*n* = 1), and Chengde (*n* = 1), respectively. All positive sample sequences were confirmed as BKV-related sequences, which validated the accuracy of detection results of the real-time RT-PCR assay, and homology analysis of partial 3D genes revealed high identity (91.8–99.8%) to each other. A phylogenetic tree showed that all 13 BKV strains were in a monophyletic branch with other BKV reference strains from different countries, while they were more distantly related to Aichivirus A and porcine kobuvirus (Figure 3). A total of 13 partial 3D sequences of BKV from Hebei had been submitted to GenBank under accession numbers: ON323472 and ON045142-ON045153.

## Discussion

As an emerging pathogen considered to be closely related to bovine diarrhea, BKV has been widely prevalent all over the world (23). Although there is still no conclusive evidence that BKV causes diarrhea in cattle (23), several studies indicated that the positive rate of BKV in diarrhea cattle was significantly higher than in healthy cattle, suggesting that BKV was most likely involved in the pathogenesis of diarrhea (15, 17, 19, 22). Furthermore, BKV had been detected as a single pathogen in cattle with diarrhea, which indicated that it is likely to be predominant in diarrhea (20, 30). Therefore, monitoring the transmission of BKV has become crucial. In contrast to the time-consuming conventional RT-PCR (1), real-time RT-PCR has become the most efficient and reliable method for monitoring the prevalence of the pathogen. Real-time RT-PCR has been widely used for early diagnosis and epidemiological surveillance of major outbreaks such as SARS-CoV2 in humans (31), African swine fever (ASF) (32), and foot-and-mouth disease (FMD) (33) in animals.

Because BKV is worldwide distributed in the cattle industry and there is potential for huge economic losses, it is necessary to develop a real-time RT-PCR assay for specific and sensitive detection of BKV. To our knowledge, a TaqMan real-time RT-PCR method had been used in the investigation of BKV in the United States (20), but this laboratory did not provide the details of the assay such as primers and probe sequences. In China, an Eva Green-based real-time PCR for BKV was reported in 2021 (34), but the fluorescent dye-based real-time PCR assay was highly prone to false positives, resulting in poor specificity. Another study by the same authors (35) reported the development of a new TaqMan probe-based assay for BKV. However, the plasmid DNA was used as a template for performance analysis, ignoring the fact that BKV is an RNA virus. In this study, *in vitro* transcribed RNA of BKV 3D gene was used as a template in the development of the real-time RT-PCR assay, and the performance of the developed assay could be evaluated more reliably. Furthermore, considering the worldwide distribution of different BKV strains, we fully compared the sequences of BKV strains available in the GenBank and identified a conserved region in the 3D gene as the amplification target. The designed primers and probe had only 0–3 base mismatches with the reference strains of the United States, United Kingdom, Canada, Japan, and Egypt (Accession numbers: MN336260, KT003671, MW605074, AB084788, KY407744). Thus, theoretically, the developed real-time RT-PCR assay enables to achieve effective detection of different BKV isolates with good specificity, sensitivity, and reproducibility, and provides a scientific, reliable, and efficient detection method for further studies about BKV.

Since the first detection of BKV in China in 2014 (16), it has been reported to be endemic in at least eight regions of China (22), whereas there has been no evidence of a BKV epidemic in Hebei to date. Hebei province is also a major area of cattle industry development, and the knowledge of the prevalence of new pathogens such as BKV is of great importance to the industry. Therefore, the developed RT-PCR assay was applied to detect 243 bovine fecal samples collected from nine regions of the Hebei province. The results showed that BKV was positive in the seven regions of Hebei province except for Langfang and Xingtai, which proved the wide prevalence of BKV in the cattle in Hebei province. More BKV-positive samples were identified in the calves than in adult cattle, which was consistent with the previous reports (15, 17, 19, 22). Furthermore, more BKV-positive samples were collected from cattle with diarrhea symptoms, and higher levels of viral RNA were detected in diarrheic cattle, which supported the suspicion that BKV is the causative agent of bovine diarrhea. A total of 13 of the 47 positive samples from each region were sequenced based on partial 3D gene and all sequences were confirmed to be BKV and phylogenetic trees clustered together with other BKV strains, which validated the reliability of the developed assay.

In conclusion, targeting the conserved region of the 3D gene, a specific, sensitive, and reliable TaqMan-based real-time RT-PCR assay for BKV was developed and evaluated on the bovine fecal samples. The reliability and accuracy of the assay were confirmed by the sequencing and phylogenetic analysis of BK- positive clinical samples. The developed real-time RT-PCR assay would be of great significance for the epidemiological investigation and effective control of BKV infection in the cattle industry.

## Data availability statement

The datasets presented in this study can be found in online repositories. The names of the repository/repositories and accession number(s) can be found below: https://www.ncbi.nlm.nih.gov/genbank/, ON323472; https://www.ncbi.nlm.nih.gov/genbank/, ON045142-ON045153.

## Author contributions

JiaW and YL conceptualized and designed the experiments. YL and LL collected the samples and performed the experiments. JinW, TW, YG, XS, and WY collected samples and analyzed the data. JiaW and RL wrote and revised the manuscript. All authors contributed to the article and approved the submitted version.

## Funding

This work was supported by the Science and Technology Program of Hebei Province (19226636D) and the Precision Animal Husbandry Discipline Group Construction Project of Hebei Agricultural University (1090064).

## Conflict of interest

The authors declare that the research was conducted in the absence of any commercial or financial relationships that could be construed as a potential conflict of interest.

## Publisher's note

All claims expressed in this article are solely those of the authors and do not necessarily represent those of their affiliated organizations, or those of the publisher, the editors and the reviewers. Any product that may be evaluated in this article, or claim that may be made by its manufacturer, is not guaranteed or endorsed by the publisher.
